# Popcorn-like nodules in the lungs of a patient with Sjögren’s disease

**DOI:** 10.1515/rir-2026-0036

**Published:** 2026-10-22

**Authors:** Yuxia Shao, Xiaoshan Yang, Li Li, Li Wang, Yanli Yang

**Affiliations:** Department of Rheumatology and Clinical Immunology, Peking Union Medical College Hospital, Chinese Academy of Medical Sciences & Peking Union Medical College, Key Laboratory of Rheumatology and Clinical Immunology, Ministry of Education, National Clinical Research Center for Dermatologic and Immunologic Diseases, Beijing, China; Department of Rheumatology and Immunology, Binzhou People’s Hospital, Binzhou, Shandong Province, China; Department of Pulmonary and Critical Care Medicine, Peking Union Medical College Hospital, Chinese Academy of Medical Sciences & Peking Union Medical College, Beijing, China

A 65-year-old woman presented with a 15-year history of Sjögren’s syndrome (SS), now termed Sjögren’s disease,^[1]^ originally diagnosed due to dry mouth and dry eyes. Hydroxychloroquine treatment was irregular. Ten years prior, a routine examination incidentally revealed multiple pulmonary nodules, showing gradual enlargement. In May 2019, she presented to Peking Union Medical College Hospital (PUMCH) with chest pain. Physical examination revealed fine crackles at the right lung base. Laboratory investigations showed: IgG 23.11 g/L (normal 7–17), urinary kappa light chain of 11.2 mg/dL (normal 0–5.1). Antinuclear antibody (ANA) was 1: 1280 granular, with strongly positive anti-SSA and anti-SSB antibodies. High-resolution computed tomography (HRCT) of the lungs showed multiple bilateral nodules with popcorn-like calcifications (Figure 1, A-D). Lung biopsy revealed amorphous eosinophilic deposits, vascular wall thickening, and plasma cell infiltration (Figure 2, A and B). Congo red staining was positive, and mass spectrometry confirmed the presence of *kappa* light chains, confirming pulmonary nodular amyloid light-chain (AL) amyloidosis. Re-evaluation of pathological findings confirmed Sjögren’s disease with secondary nodular pulmonary AL amyloidosis. Subsequently treated with prednisone acetate (50 mg/d) and cyclophosphamide (50 mg/d), the patient remains stable after 5 years with no CT progression on maintenance therapy.

**Figure 1:**
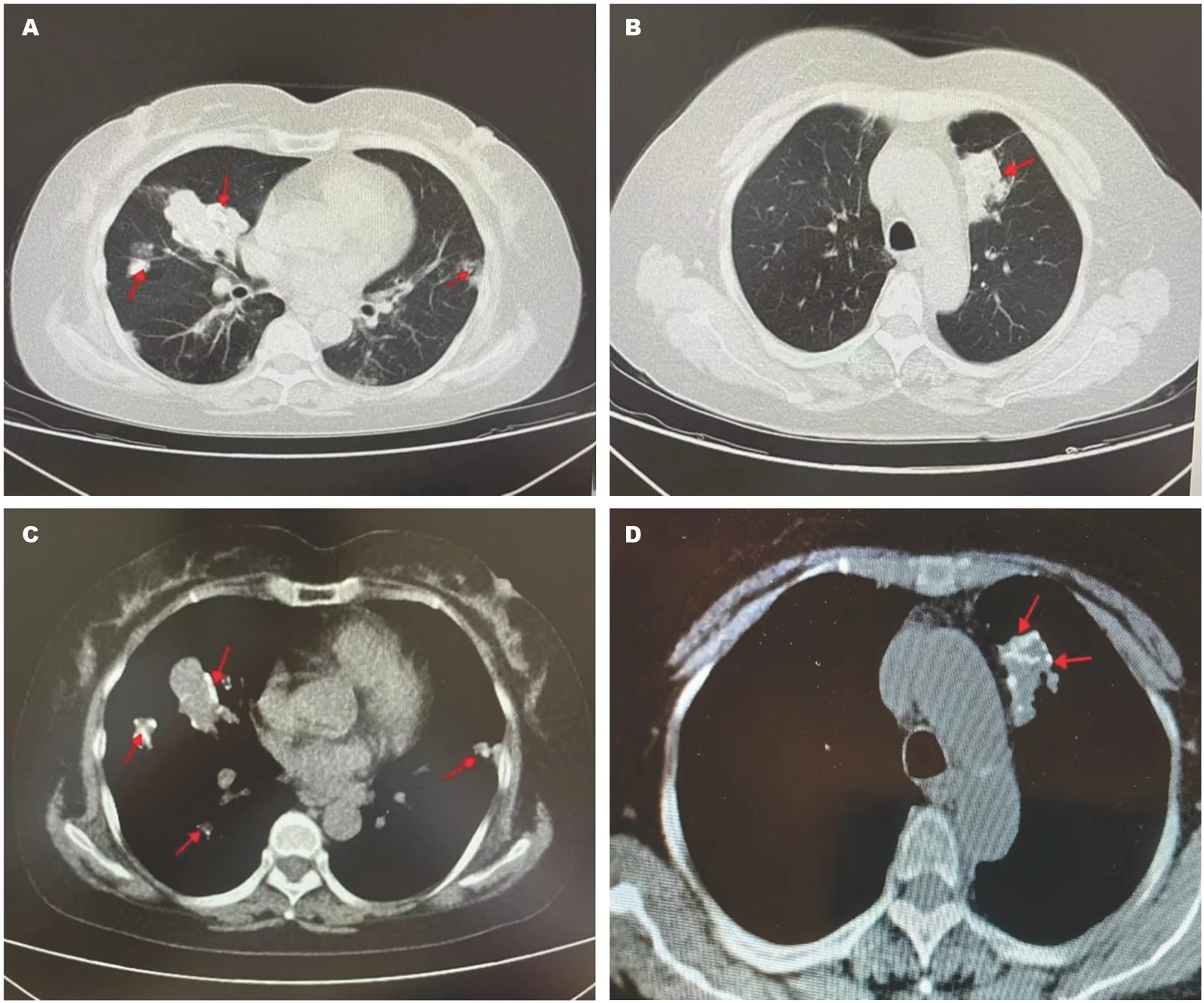
A-D (A, B) Chest computed tomography (lung windows) and (C, D) (mediastinal windows) showing bilateral pulmonary nodules with popcorn-like calcifications.

**Figure 2:**
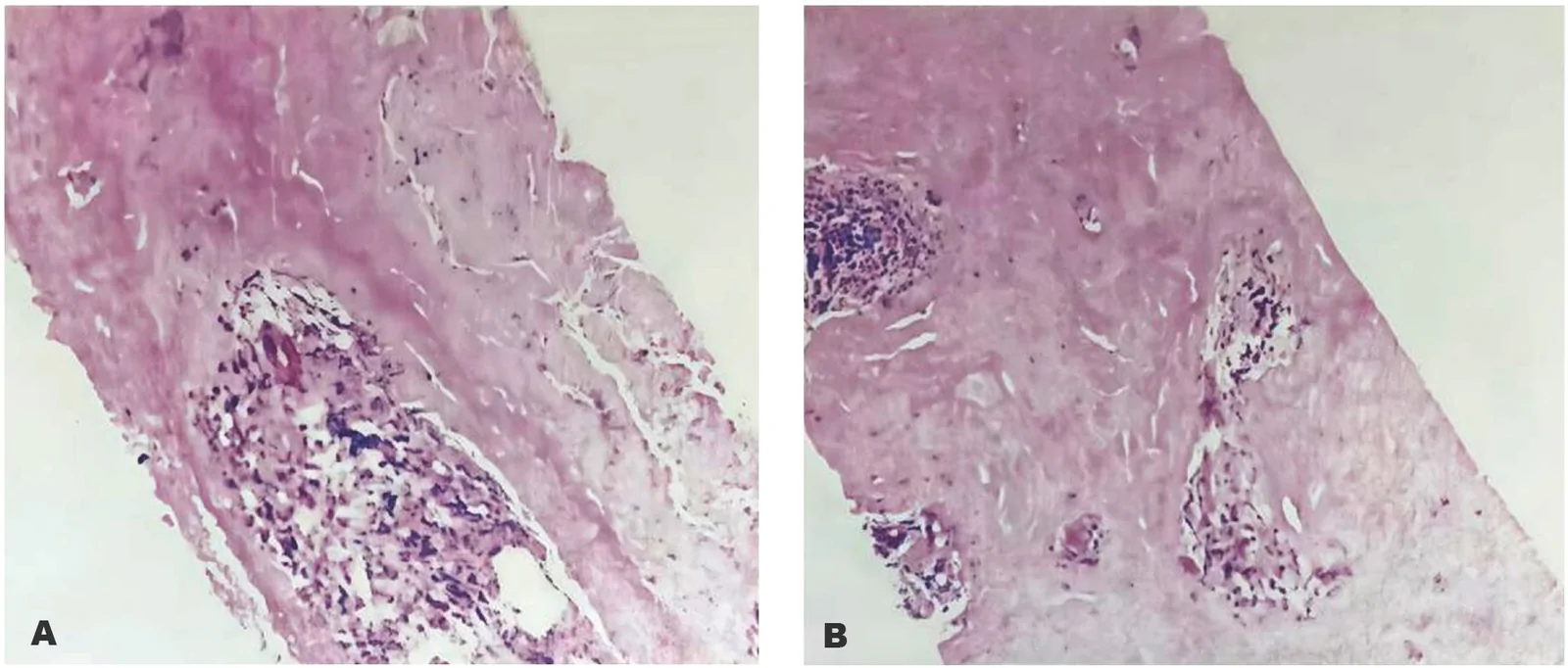
(A, B) Histopathology of lung biopsy showing amorphous eosinophilic deposits, vascular wall thickening, and plasma cell infiltration. Special staining confirmed amyloidosis.

Nodular pulmonary amyloidosis is a rare manifestation of localized AL amyloidosis. In Sjögren’s disease, plasma cell infiltration can lead to immunoglobulin light chain deposition and amyloid fibril formation.^[2]^ Differential diagnoses for multiple calcified pulmonary nodules include malignancies (*e.g*., lung cancer, lymphoma) and infections (*e.g*., tuberculosis, fungal infections).^[3]^ However, the combination of multiple nodules with classic popcorn-like calcifications on CT and positive Congo red staining strongly suggests amyloidosis.^[4]^ This case highlights that pulmonary nodular amyloidosis should be considered in patients with long-standing Sjögren’s disease presenting with multiple calcified pulmonary nodules. The primary goal is to control underlying autoimmunity with glucocorticoids and disease-modifying antirheumatic drugs. While targeted therapies like rituximab are options,^[4]^ our patient achieved stability in clinical symptoms, pulmonary function (Table 1, A and B), and imaging, necessitating long-term follow-up.

**Table 1:** Pulmonary function test results.

Parameter	Unit	Predicted value	Measured value	% Predicted
A. Pulmonary ventilation function				
Maximal vital capacity	L	2.76	2.76	100
FEV_1_	L	2.22	1.98	89
FVC	L	2.65	2.75	104
FEV_1_%FVC	%	83.97	71.87	86
FEV_1_%VC max	%	77.13	71.53	93
FEV_1_×30	%	88.36	59.31	67
Extrapolated volume	L	–	0.07	–
B. Diffusion function				
VA	L	4.75	4.06	86
DLCO SB	mL/min/mmHg	7.42	4.97	67
DLCO/VA	mL/min/mmHg/L	1.51	1.22	81
Hemoglobin	g/dL	–	13.40	–
DLCOc SB	mL/min/mmHg	7.42	4.97	67
DLCOc/VA	mL/min/mmHg/L	1.51	1.22	81

FEV_1_, forced expiratory volume in 1 second; FVC, forced vital capacity; VC max, maximal vital capacity; VA, alveolar volume; DLCO SB, single-breath diffusing capacity of the lung for carbon monoxide; DLCOc SB, corrected DLCO for hemoglobin.
